# An 8 MeV Electron Beam Modified In:ZnO Thin Films for CO Gas Sensing towards Low Concentration

**DOI:** 10.3390/nano11113151

**Published:** 2021-11-22

**Authors:** Aninamol Ani, P. Poornesh, Albin Antony, K. K. Nagaraja, Ashok Rao, Gopalkrishna Hegde, Evgeny Kolesnikov, Igor V. Shchetinin, Suresh D. Kulkarni, Vikash Chandra Petwal, Vijay Pal Verma, Jishnu Dwivedi

**Affiliations:** 1 Department of Physics, Manipal Institute of Technology, Manipal Academy of Higher Education, Manipal 576104, Karnataka, India; aninamol.ani@learner.manipal.edu (A.A.); albin.antony007@learner.manipal.edu (A.A.); nagaraja.kk@manipal.edu (K.K.N.); a.rao@manipal.edu (A.R.); 2 Department of Functional Nanosystems and High-Temperature Materials, National University of Science and Technology “MISiS”, Leninskiy Pr. 4, 119049 Moscow, Russia; kolesnikov.ea@misis.ru; 3 Department of Physical Materials Science, National University of Science and Technology “MISiS”, Leninskiy Pr. 4, 119049 Moscow, Russia; ingvar@misis.ru; 4 Department of Nano-Sciences, Indian Institute of Science, Bengaluru 560012, Karnataka, India; gopalkrishna@iisc.ac.in; 5 Department of Atomic and Molecular Physics, Manipal Academy of Higher Education, Manipal 576104, Karnataka, India; suresh.dk@manipal.edu; 6 Industrial Accelerator Section, PSIAD, Raja Ramanna Centre for Advanced Technology, Indore 452012, Madhya Pradesh, India; vikash@rrcat.gov.in (V.C.P.); vijaypal@rrcat.gov.in (V.P.V.); jishnu@rrcat.gov.in (J.D.)

**Keywords:** indium-doped ZnO, electron beam irradiation, CO gas sensing

## Abstract

In the present investigation, electron beam-influenced modifications on the CO gas sensing properties of indium doped ZnO (IZO) thin films were reported. Dose rates of 5, 10, and 15 kGy were irradiated to the IZO nano films while maintaining the In doping concentration to be 15 wt%. The wurtzite structure of IZO films is observed from XRD studies post electron beam irradiation, confirming structural stability, even in the intense radiation environment. The surface morphological studies by SEM confirms the granular structure with distinct and sharp grain boundaries for 5 kGy and 10 kGy irradiated films whereas the IZO film irradiated at 15 kGy shows the deterioration of defined grains. The presence of defects viz oxygen vacancies, interstitials are recorded from room temperature photoluminescence (RTPL) studies. The CO gas sensing estimations were executed at an optimized operating temperature of 300 °C for 1 ppm, 2 ppm, 3 ppm, 4 ppm, and 5 ppm. The 10 kGy treated IZO film displayed an enhanced sensor response of 2.61 towards low concentrations of 1 ppm and 4.35 towards 5 ppm. The enhancement in sensor response after irradiation is assigned to the growth in oxygen vacancies and well-defined grain boundaries since the former and latter act as vital adsorption locations for the CO gas.

## 1. Introduction

Carbon Monoxide (CO) is a colorless, odorless, and tasteless gas, making it enormously dangerous to human life [1,2]. It is mainly produced by reason of partial combustion of fuels containing carbon and is predominantly found during burning of domestic fuels, in automobile exhausts, power plants, and accidental leakage from home appliances, such as heaters, etc. [3]. Due to its properties, it is often difficult to detect the presence of CO, describing it as a ‘silent killer’ [4]. The inhalation of CO can deprive the capacity of blood to carry oxygen and combines with hemoglobin to form carboxyhemoglobin [5]. Further, it results in the misfunctioning of all vital organs in the body, which may eventually be fatal [6]. The U.S. Environmental Protection Agency (EPA) commends an atmospheric air standard of 9 ppm CO over 8 h and 35 ppm over 1 h [7]. In addition, different people have different tolerance to the same CO concentration level. Therefore, the early monitoring of CO at a low concentration itself is significant from the safety standpoint.

Several researches have been carried out by scientists employing various types such as resistive, field effect transistor, optical, acoustic, and electrochemical based gas sensors, among others, to identify the presence of CO gas in the surroundings where it is expected to be present [8,9,10,11,12]. However, resistive-based gas sensors, also known as metal oxide semiconductor (MOS) gas sensors, offer considerable advantages over other types due to the facile fabrication, less complexity in structure, simple operation, low production cost, and miniaturization [13,14]. Also, MOS gas sensors are broadly accepted around the globe owing to their high sensitivity and selectivity towards majority of toxic gases, including CO, CO_2_, SO_2_, H_2_S, etc. [15,16,17,18]. Some of the widely used MOS gas sensors are ZnO, WO_3_, SnO_2_, etc. [19,20,21]. In particular, ZnO is an interesting material for gas sensing due to its distinctive properties, including intrinsic n-type conductivity, chemical and thermal stability, and so on [22,23].

In a previous study, we explored the role of indium (In) doping to ZnO thin films prepared by means of the low-cost spray pyrolysis technique in tuning CO gas sensing properties [24]. It was found that 15 wt% indium doped ZnO (IZO) films exhibited better sensing characteristics towards lower CO concentrations, particularly 1 ppm (response = 1.84) compared to pristine, 5 wt%, and 10 wt% films [24]. At present, a post-treatment approach is adopted to further tune CO sensing characteristics. The 15 wt% IZO films are irradiated with electron beam at various dosages and the resulting characteristics are studied. Electron beam irradiation is a well-known technique for modifying the morphology and physicochemical properties of a material [25,26,27]. The applicability at room temperature and ease of manipulation makes it a convenient method for altering the material’s properties [26,27].

Jae-Hun Kim et al. [26] studied the effect of electron beam irradiation on nano-fibred ((NFs) ZnO) for H_2_ detection. They noticed the formation of surface defects upon irradiation and the nanofiber irradiated with 150 kGy electron beam of energy 1 MeV exhibited excellent H_2_ sensing performance. The group also studied the H_2_ sensing ability of Pd filled ZnO NFs and 150 kGy irradiated NF showed a better response of 74.6–100 ppb H_2_ at a temperature of 350 °C [28]. Vattappalam et.al. [29] subjected the silar synthesized Al:ZnO thin films to electron beam irradiation with dose rates 6 and 8 kGy. The average sensitivity towards ethanol vapour is found to be 0.50 and 0.60 for unirradiated and irradiated thin films. Close inspection of the literature reveals that the electron beam treated IZO films prepared via low-cost spray pyrolysis for modifying the CO sensing performance is seldom reported. Besides, the CO sensing by resistive-based gas sensors, particularly towards low concentration below 5 ppm, is not explored well. The low detection limit is of extreme importance by which the ZnO material can be further tuned for serum detection in medical fields apart from gas sensing [30]. In the present study, the sensing of CO concentrations below 5 ppm is detected employing IZO thin films treated with electron beam irradiation as the sensing layer.

## 2. Particulars of the Experiment

### 2.1. Synthesis of IZO Thin Films and Electron Beam Treatment

Indium-doped ZnO thin films were deposited on soda lime glass substrates by spray pyrolysis method. Zinc acetate dihydrate ((CH_3_COO)_2_ Zn·2H_2_O) (Merck Life Science Pvt.Ltd., Mumbai, MH, India) and indium (III) nitrate hydrate (InN_3_O_9_·xH_2_O) (Merck Life Science Pvt. Ltd., Mumbai, MH, India) were used as host and dopant precursor solutions, respectively. The molarity of the host solution was maintained to be 0.05 M and acetic acid (CH_3_COOH) (Merck Life Science Pvt.Ltd., Mumbai, MH, India) was added in drops to avoid any precipitation and stirred magnetically to obtain a homogeneous solution. The dopant precursor is added to the host for achieving the desired concentration of 15 wt% with indium doping. The temperature of the substrate was maintained at 425 °C. The thickness profilometer was used to measure the film thickness and it was around 300 nm.

**Figure 1:**
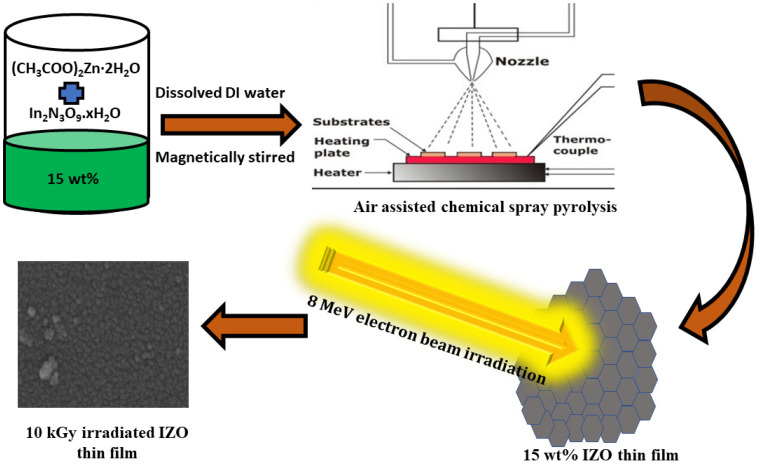
Schematic of IZO thin films preparation and electron beam irradiation.

The electron beam irradiation on IZO thin films was performed at atmospheric conditions by the use of Linear Accelerator (LINAC) at Raja Ramanna Centre for Advanced Technology, Indore, India. The energy of the e-beam is 8 MeV and the films were exposed to 5, 10, and 15 kGy dose rates with a beam current of 50 mA and power of 250 W. A schematic of the preparation and electron beam irradiation is shown in Figure 1.

### 2.2. IZO Thin Film Characterization

The crystal orientation and structural stability of e-beam irradiated IZO thin films were obtained by the Rigaku Ultima IV X-ray diffractometer (XRD) (Rigaku, Tokyo, Japan). Cu K-alpha of energy 8.04 keV corresponding to wavelength of 1.54 angstrom is the source of X-ray utilized in XRD. The optical properties were measured using a Shimadzu 1800 UV-VIS spectrophotometer (Shimadzu Corporation, Kyoto, Japan). Tescan VEGA 3 Scanning Electron Microscope (SEM) (Tescan, Brno, Czech Republic) was utilized to analyze the surface morphological characteristics of the grown IZO thin films. The study of defects in the irradiated films, specifically oxygen vacancies from a gas sensing point of view, is carried out by Photoluminescence (PL) spectroscopy using Jasco fp-8300spectrofluorometer (JASCO Corporation, Tokyo, Japan). X-ray photoelectron spectroscopy (XPS) was implemented via Kratos Axis Ultra DLD XPS Spectrometer (Kratos, Manchester, UK) to inspect the chemical composition of the films.

### 2.3. Gas Sensing Tests

The gas sensor measurement setup was used to record the readings of resistance variation in the existence of dry air and CO gas. Mass flow controllers (MFCs) managed the flow of the gases and the total flow was maintained at 500 sccm. Initially, the dry air composed of 80% nitrogen and 20% oxygen was passed to the closed gas chamber where the sensor is kept and the value of resistance was noted from 2450 source meter of Keithley. The CO gas being the target gas was mixed with dry air in required concentrations and made to flow to the chamber. The variation in resistance after contact with the CO gas was measured. The response time, recovery time, and response were calculated from the obtained measurements.

## 3. Results and Discussion

### 3.1. Structural Investigation

**Figure 2:**
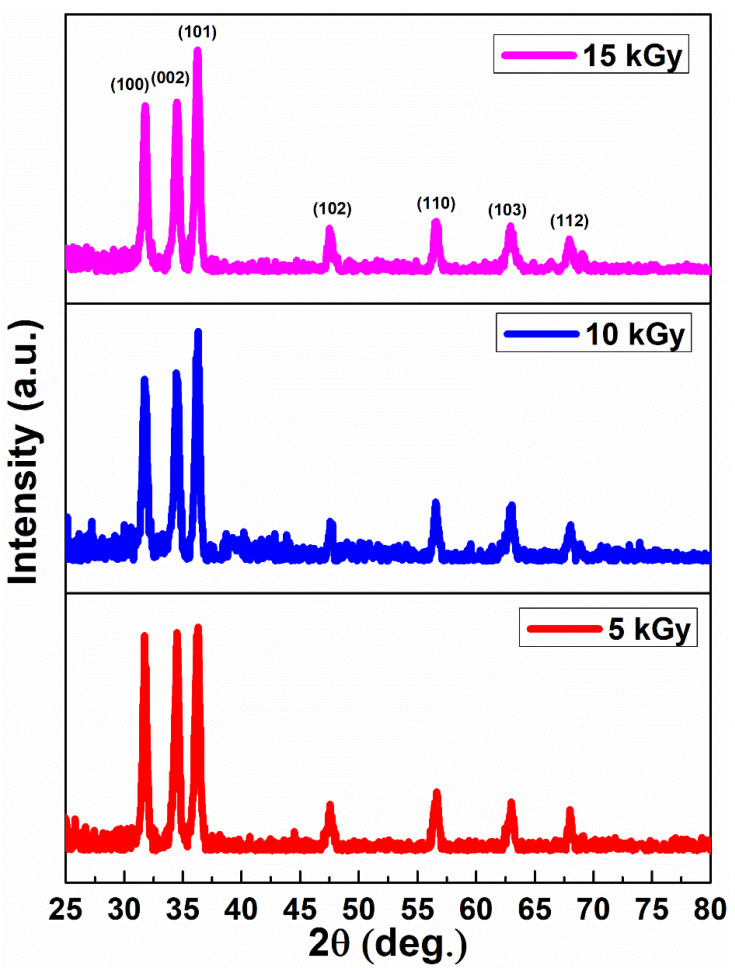
XRD pattern of irradiated IZO thin films.

The polycrystalline nature and the hexagonal ZnO with wurtzite structure of the unirradiated and irradiated films are evident from the XRD pattern shown in Figure 2. The diffraction peaks belong to space group P63mc corresponding to (100), (002), (101), (102), (110), (103), and (112) planes of ZnO with JCPDS card file 36–1451 [31]. The (101) plane is the most intense compared to other planes. For the unirradiated 15 wt% IZO film data, refer back to our previous study [24].

The structural parameters, namely crystalline size (*D*), dislocation density (*δ*), and strain (*ε*) is given by the following equations [32,33]:

(1) $$D=\frac{K\lambda }{\beta \cos\theta }$$

(2) $$\delta =\frac{1}{D^{2}}$$

(3) $$\varepsilon =\frac{\beta \cos\theta }{4}$$

where *k* is the shape factor with value of 0.9, *θ* is the glancing angle, *λ* is the X-ray wavelength *o,* and *β* is the full width half maximum. The obtained values are tabulated in Table 1.

The obtained XRD results reveal that there are minimal variations in the structural parameters. This approves that the IZO films display uniform structure on various irradiation dosages, thereby exhibiting excellent structural stability, even in the powerful radiation atmosphere.

### 3.2. Morphological Properties

**Figure 3:**
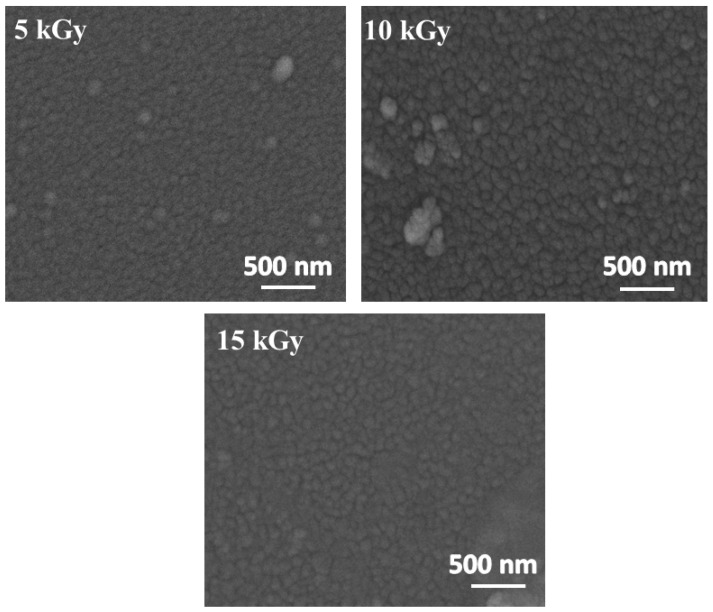
SEM images of irradiated IZO thin films.

SEM characterization was executed to study the morphological surface features of the irradiated IZO thin films. Figure 3 represents the SEM images of 5 kGy, 10 kGy, and 15 kGy irradiated IZO thin films. For the SEM image of unirradiated 15 wt% IZO thin film, refer back to [24].

It is observed that there was no substantial change in morphology when irradiated with 5 kGy, indicating the dose rate was insufficient to produce any variations. However, a nominal but visible change is observed in 10 kGy irradiated IZO film with well-defined grain boundaries. From a practical standpoint of gas sensing, grain boundaries being a two-dimensional defect serve as a dynamic adsorption spot for target gas, thereby enhancing the sensing performance [26]. Further increase in e-beam dosage of 15 kGy lead to the commencement of degradation of grain boundaries unfavorable for the mechanism of sensing.

### 3.3. Photoluminescence Analysis

The IZO thin films possess intrinsic point defects such as oxygen or Zn vacancies, Zn or oxygen interstitials, etc. [34]. Also, the irradiation of the films with electron beam can cause changes in the defects or introduce any defects into crystal lattice [35]. RTPL spectra at an excited wavelength of 325 nm is recorded to obtain the various emissions and Gaussion deconvolution is performed to identify the individual defects responsible for the corresponding emissions [34,35].

**Figure 4:**
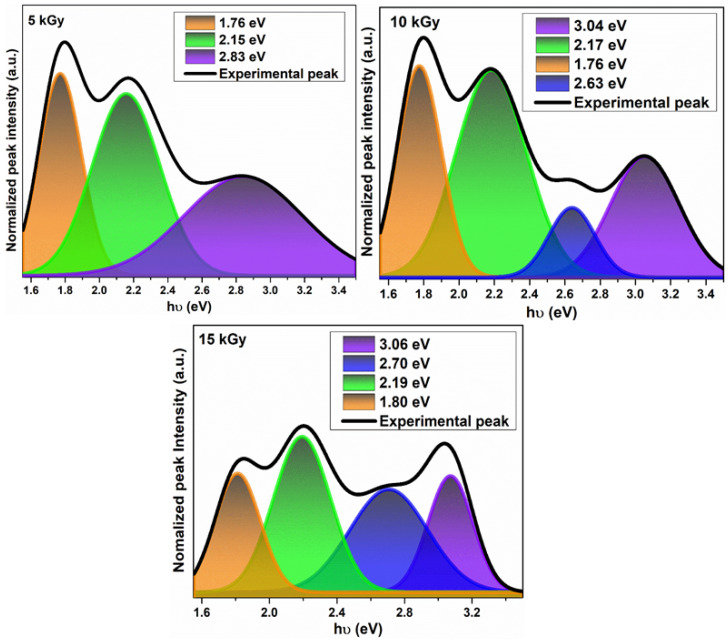
RTPL spectra of irradiated IZO thin films deconvoluted by Gaussian function.

The RTPL spectra of irradiated IZO films is shown in Figure 4.

The emission around 2.96 eV–2.88 eV represents the near band edge emission corresponding to the exciton–exciton collision, resulting in free exciton recombination [36,37]. The blue and orange emissions correspond to oxygen interstitial defects. The green emission represents the oxygen vacancy defects, which is one among the factors responsible for gas sensing [34,35,36,37]. The FWHM of the green emissions are 0.42, 0.45, and 0.39 for 5, 10, and 15 kGy irradiated IZO films, respectively. The FWHM is higher for 10 kGy irradiated, supporting the fact that the oxygen vacancies are enhanced in the film after irradiation. The blue emission is absent in IZO film irradiated at 15 kGy and an additional yellow emission at 1.88 eV corresponding to oxygen interstitial is observed [36].

### 3.4. XPS Investigations

**Figure 5:**
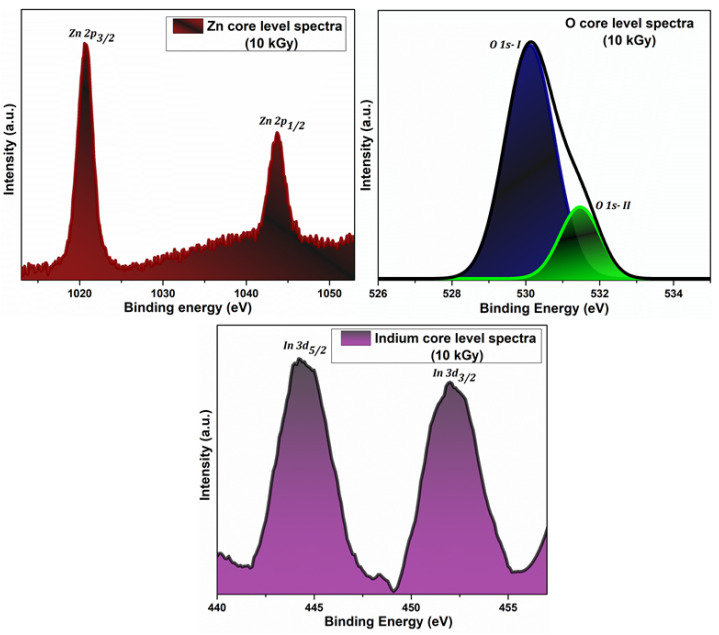
XPS core level spectra of Zn, O, and In.

The distinct occurrence of Zn, O, and In in IZO films were confirmed from XPS spectra, thereby revealing the chemical composition. The Zn, O, and In core level spectra of 10 kGy irradiated 15 wt% IZO film is shown in Figure 5. For the XPS spectra of unirradiated 15 wt% IZO film, refer back to [24].

The binding energies observed at 1020.6 eV and 1043.7 eV corresponds to Zn 2p_3/2_ and Zn 2p_1/2_ peaks, respectively. The +2 oxidation state of the Zn is confirmed from the obtained energy values for Zn [38,39]. The core level spectra of oxygen are deconvoluted by Gaussian function to identify the individual components, namely O 1s-I and O 1s-II. The O 1s-I peak at 530.1 eV and O 1s-II peak at 531.48 eV represents O^2−^ ions shared with Zn atom and oxygen vacancy defects, respectively [38,39]. The binding energy at the doublet peaks of 444 eV and 452 eV signifies the In 3d_5/2_ and In 3d_3/2_ states, respectively, thereby confirming the +3 oxidation state of In. The results obtained for Zn, O, and In suggest the successful chemical stability of IZO thin film post electron beam irradiation [40].

### 3.5. CO Sensing Studies

Since gas adsorption-desorption is the process taking place in the sensing mechanism, it is essential to optimize the operating temperature [26]. Hence, the optimal operating temperature is considered to be 300 °C and sensing voltage is set at 1 V.

Initially the dry air is passed to the chamber, which results in oxygen adsorption by the irradiated sensor. Anions of oxygen such as O_2_^−^, O^−^ and O^2−^ are formed during the process of extraction of electron from the conduction band by the adsorbed oxygen, leaving a layer depleted of electrons with high resistance. The formation of oxygen anions are as followed by the reactions [41]:

O_2_ (g) → O_2_ (ads) (4)

O_2_ (ads) + e^−^ ↔ O_2_^−^ (ads) (5)

O_2_^−^ (ads) + e^−^ ↔ 2O^−^ (ads) (6)

Once the sensor resistance is stabilized, the CO gas is purged. The sensing layer comes into contact with the CO gas molecules and the electrons are sent back to the conduction band. The surface reactions on exposure to CO gas are given by [42]:

CO + O^−^ ads ↔ CO_2_ + e^−^ (7)

2CO + O_2_^−^ ads ↔ 2CO_2_ + e^−^ (8)

**Figure 6:**
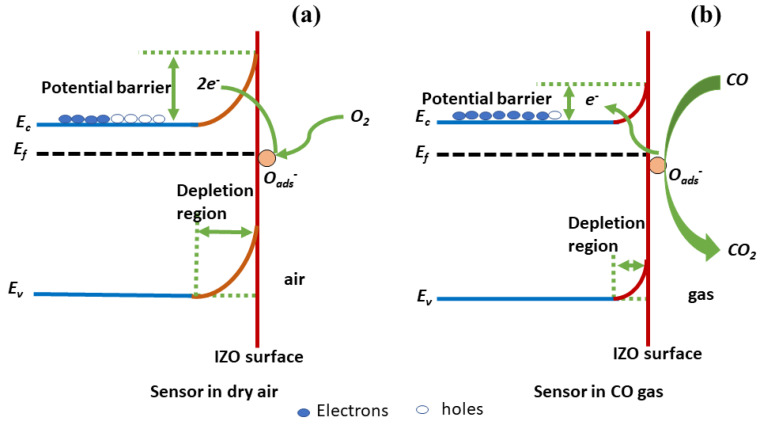
Band diagram of the proposed sensing mechanism (**a**) Sensor in dry air (**b**) Sensor in CO gas.

This results in decrease in the resistance and such changes in resistance values are recorded for extracting the sensing parameters [13,43]. The entire process described is exclusive to the IZO sensor being n-type [44] and CO gas being reducing in nature [45]. Figure 6 shows the band diagram of the proposed sensing mechanism.

**Figure 7:**
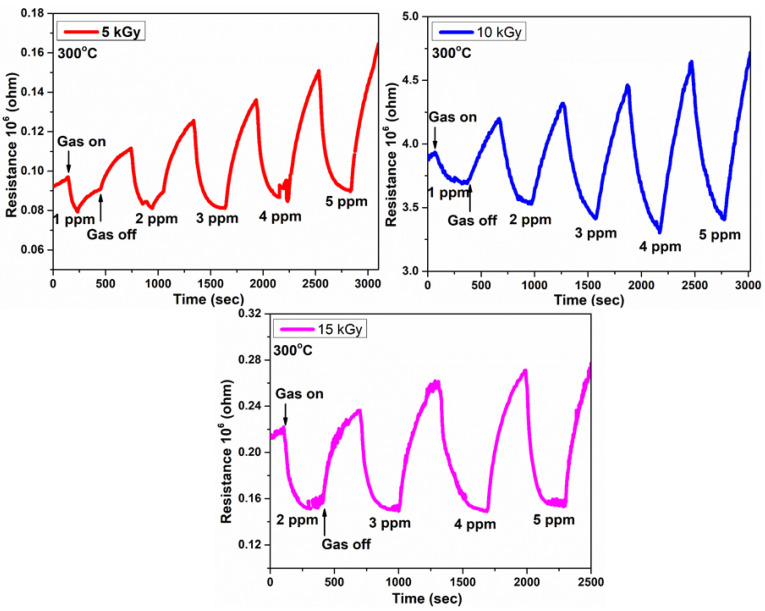
Sensing response curves of e-beam treated IZO thin films.

The sensing response curves of e-beam treated IZO thin films are shown in Figure 7.

The 5 and 10 kGy irradiated films were able to detect the CO concentration lower to 1 ppm whereas 15 kGy irradiated film could not detect 1 ppm. The response time and recovery time are calculated and given in Table 2.

The 10 kGy irradiated film exhibited quick response time and recovery time towards various concentrations of CO gas. It exhibited a short response time of 15 s towards 1 ppm of CO gas and 52 s towards 5 ppm of CO gas. The recovery time for 1 ppm of CO gas was found to be 29 s and 43 s for 5 ppm of CO gas. The sensor response is defined as the ratio of resistance of the sensor in dry air to the ratio of resistance in the CO gas, calculated by the formula [15]:

Response = (R_a_/R_g_) (9)

Table 3 shows the response towards CO gas by irradiated films.

**Figure 8:**
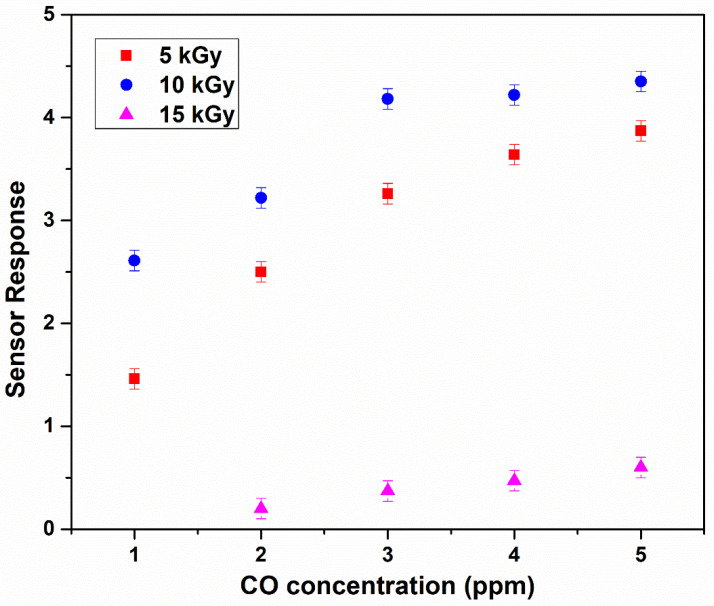
Calibration curve corresponding to Figure 7 (error is ±0.1).

It exhibited a response of 2.61 and 4.35 towards low concentrations of 1 ppm and 5 ppm of CO gas, respectively. The high response can be ascribed to the enhanced oxygen vacancies and large grain boundaries observed from RTPL and SEM analysis, respectively. Both grain boundaries and oxygen vacancies act as a mobile adsorption site for the CO gas molecules [26]. It was found that beyond 10 kGy i.e., for 15 kGy irradiated IZO film, the sensing performance was significantly reduced. This could be supported by the degraded surface morphology and quench in oxygen vacancies at 15 kGy dose rate observed from SEM and RTPL, respectively. The calibration curve corresponding to Figure 7 is shown in Figure 8.

Selectivity is an important parameter from a practical standpoint. In our previous study [19], we studied CO sensing properties e-beam treated ZnO thin films. We also studied the CO sensing properties of unirradiated IZO thin films [24]. Both of our previous studies reveal that the irradiated ZnO and unirradiated IZO films exhibit excellent selectivity towards CO gas in comparison to NO_2_, SO_2_, and H_2_S. Since a selective nature towards CO was shown by irradiated ZnO and unirradiated IZO films, we further investigated the CO gas sensitivity exhibited by irradiated IZO thin films.

Table 4 shows a comparison study of CO gas sensing performance of electron beam irradiated IZO thin films with other recent studies.

## 4. Conclusions

ZnO thin films doped with 15 wt% indium synthesized through low-cost spray pyrolysis technique were subjected to e-beam irradiation at 5, 10, and 15 kGy dose rates. The structural stability of the irradiated films was maintained and confirmed from XRD studies. The surface morphology obtained from SEM revealed that 10 kGy irradiated film has better characteristics such as well-defined grain boundaries required for the gas adsorption mechanism. The presence of defects preferably oxygen vacancies in terms of gas sensing was confirmed from PL studies. The dose rate of 10 kGy was found to be the optimum rate in introducing adequate amount of oxygen vacancies for exhibiting better sensing with a response of towards 1 ppm, compared to other irradiated films. XPS studies assured the chemical composition of IZO film with Zn, O, and In as the individual elements. The 15 wt% IZO thin film irradiated at a dose rate of 10 kGy exhibited a better response of 2.61 towards 1 ppm and 4.35 towards 5 ppm at 300 °C, compared to other irradiated films. The melioration in oxygen vacancy defects and well-defined grain boundaries are responsible for the enhanced CO sensing. Hence, electron beam irradiation could be used as a convenient means to modify the physicochemical properties of IZO thin films for improving the CO sensing characteristics. Also, the electron beam treatment may serve as a probable way to change the characteristics of any metal oxide semiconductor material suitable for gas sensing performance.

## Figures and Tables

**Table 1:** Values of structural variables of irradiated IZO thin films.

Dosage	Crystalline Size, *D* (nm)	Dislocation Density, *δ* (10^15^ Lines m^−2^)	Strain, *ε* (10^−3^)
5 kGy	18.87	2.82	1.84
10 kGy	18.40	2.94	1.87
15 kGy	18.97	2.81	1.83

**Table 2:** Response time and recovery time of irradiated IZO sensors.

	15 wt% In Doped ZnO					
	Response Time (s)			Recovery Time (s)		
Irradiation Dosage (kGy)/Gas Conc. (ppm)	5	10	15	5	10	15
1	74	15	106	240	29	57
2	48	25	100	161	80	93
3	54	66	108	116	68	100
4	39	46	102	79	53	83
5	32	52	82	61	43	91

**Table 3:** Response of irradiated IZO sensors.

Irradiation Dosage (kGy)/Gas Conc. (ppm)	Sensor Response (15 wt% In Doped ZnO) (Response ±0.10)		
	5	10	15
1	1.46	2.61	-
2	2.50	3.22	0.20
3	3.26	4.18	0.37
4	3.64	4.22	0.47
5	3.87	4.35	0.60

**Table 4:** Comparison study.

Material	Preparation Method	Conc. (ppm)	Operating Temperature	Sensor Response	Reference
Electron beam irradiated (10 kGy) IZO thin films	Spray Pyrolysis	5 (LDL:1 ppm)	300 °C	4.35 (2.61 (1 ppm))	This work
In:ZnO nanoparticles	sol-gel	50 (LDL:5)	300 °C	4.80	[46]
In:ZnO nanoparticles	Sol-gel	50 (LDL:5)	300 °C	3.5	[47]
Cu:ZnO	Co-sputtering	20	350 °C	2.7	[48]
Al:ZnO nanoparticles	Sol-gel	50 (LDL:5)	300 °C	1.6	[7]
ZnO:rGO nanoparticles	hydrothermal	1000	200 °C	7	[49]

## Data Availability

The data presented in this study are available on request from the corresponding author.
